# Nature and mechanisms of aluminium toxicity, tolerance and amelioration in symbiotic legumes and rhizobia

**DOI:** 10.1007/s00374-018-1262-0

**Published:** 2018-02-12

**Authors:** Sanjay K. Jaiswal, Judith Naamala, Felix D. Dakora

**Affiliations:** 1 Department of Chemistry, Tshwane University of Technology, Arcadia campus, 175 Nelson Mandela Drive, Private Bag X680, Pretoria, 0001 South Africa; 2 Department of Crop Sciences, Tshwane University of Technology, Arcadia campus, 175 Nelson Mandela Drive, Private Bag X680, Pretoria, 0001 South Africa

**Keywords:** Nitrogen fixation, Abiotic stress, miRNA, Acid soils, Rhizosphere exudation, Efflux pumps

## Abstract

Recent findings on the effect of aluminium (Al) on the functioning of legumes and their associated microsymbionts are reviewed here. Al represents 7% of solid matter in the Earth’s crust and is an important abiotic factor that alters microbial and plant functioning at very early stages. The trivalent Al (Al^3+^) dominates at pH < 5 in soils and becomes a constraint to legume productivity through its lethal effect on rhizobia, the host plant and their interaction. Al^3+^ has lethal effects on many aspects of the rhizobia/legume symbiosis, which include a decrease in root elongation and root hair formation, lowered soil rhizobial population, and suppression of nitrogen metabolism involving nitrate reduction, nitrite reduction, nitrogenase activity and the functioning of uptake of hydrogenases (Hup), ultimately impairing the N_2_ fixation process. At the molecular level, Al is known to suppress the expression of nodulation genes in symbiotic rhizobia, as well as the induction of genes for the formation of hexokinase, phosphodiesterase, phosphooxidase and acid/alkaline phosphatase. Al toxicity can also induce the accumulation of reactive oxygen species and callose, in addition to lipoperoxidation in the legume root elongation zone. Al tolerance in plants can be achieved through over-expression of citrate synthase gene in roots and/or the synthesis and release of organic acids that reverse Al-induced changes in proteins, as well as metabolic regulation by plant-secreted microRNAs. In contrast, Al tolerance in symbiotic rhizobia is attained via the production of exopolysaccharides, the synthesis of siderophores that reduce Al uptake, induction of efflux pumps resistant to heavy metals and the expression of metal-inducible (*dme*RF) gene clusters in symbiotic Rhizobiaceae. In soils, Al toxicity is usually ameliorated through liming, organic matter supply and use of Al-tolerant species. Our current understanding of crop productivity in high Al soils suggests that a much greater future accumulation of Al is likely to occur in agricultural soils globally if crop irrigation is increased under a changing climate.

## Introduction

Food legumes contribute significantly to human diets, especially of poor people around the world. Legumes, therefore, play a major role in reducing poverty, improving human health and nutrition and enhancing ecosystem functioning. With more than 78.3 million ha of land planted to legumes, these species provide over 35% of the world’s protein intake (Werner and Newton 2005; http://www.fao.org/).

Uniquely, legumes together with *Parasponia* (Lafay et al. 2006) are the only plant species that can form root nodules with soil rhizobia and convert atmospheric N_2_ into NH_3._ Biological nitrogen fixation (BNF) by legumes is therefore a major source of N for agriculture (Zahran 1999) and is the most important biological process on Earth, after photosynthesis and organic matter decomposition (Unkovich et al. 2008). As a result, BNF is the most critical and key process to sustainable land management, especially where N is the nutrient limiting crop production (Hungria and Vargas 2000). The legume-rhizobia symbiosis is therefore the most important contributor of symbiotic N in natural and agricultural ecosystems, as it accounts for approximately 80% of biologically fixed N in agricultural systems (Zahran 1999). According to Herridge et al. (2008), N_2_-fixing plants contribute approximately 50–70 million t of biologically fixed N annually to agricultural systems, of which 12–25 million t come from pasture and fodder legumes, 5 million t from rice, 0.5 million t from sugar cane, < 4 million t from non-legume crop land and < 14 million t from existing savannas. However, the amount of N fixed can vary between species and locations due to differences in soil factors, legume genotype, rhizobial strain and cropping pattern (Dakora and Keya 1997). Unlike chemical N fertilisers, BNF is a cheap, readily available and eco-friendly source of N (Dakora and Keya 1997), the use of which reduces environmental pollution (Ferreira et al. 2012).

Despite the enormous benefits of BNF to agricultural production, its exploitation has been limited by abiotic factors such as salinity, extreme temperatures and aluminium (Al) stress (Igual et al. 1997; Lima et al. 2009), which can all affect the legume host, the microsymbiont or both (Dakora and Keya 1997). Due to its widespread distribution, Al is a major constraint to crop production (Kochian et al. 2004). Approximately 50% of the world’s arable land is considered acidic with an underlying problem of Al toxicity (Kochian et al. 2015; Ligaba et al. 2004; Lin et al. 2012; Simões et al. 2012). In fact, Al toxicity has been reported in 67% of the world’s acidic soils (Lin et al. 2012). In addition to identifying new niches for nitrogen fixation and legume production for increased food security (Unkovich et al. 2008), legumes and rhizobia should be screened for tolerance of Al stress for use in Al-rich soils (Abdel-Salam et al. 2010). This review summarises the nature and mechanisms of Al toxicity, tolerance and amelioration in symbiotic legumes and their associated bacterial symbionts.

## Nature of aluminium stress

Al is the third most abundant element, after oxygen and silicon, and forms approximately 7% of the total solid matter in soils (Arunakumara et al. 2013; Frankowski 2016; Ma et al. 2001; Roy and Chakrabartty 2000). Soil Al is either bound to ligands (Yu et al. 2012) or occurs in harmless forms such as precipitates and aluminosilicates (Ma et al. 2001; Zhou et al. 2011) and constitutes about 1 to 25% of the soil depending on the parent rock and soil type (Barabasz et al. 2002). However, under acidic conditions, mineral Al solubilises into trivalent Al^3+^, which is highly toxic to animals, plants and microbes (Ma et al. 2001; Zioła-Frankowska and Frankowski 2018). About 40% of the world’s potential arable land is already acidic; therefore, any further increase in soil acidity from anthropogenic activity and/or acid rain can only further enhance the problem of Al toxicity and reduce agricultural productivity.

## Forms of aluminium in soils

In the soil environment, Al exists mainly as inorganic, soluble and/or organic forms. Inorganic Al is exchangeable in soil but can also be bound to silicate clays, hydrous oxides, sulphates and phosphates (Violante et al. 2010). In acidic soils (pH ≤ 5.5), these mineral forms of aluminium can dissolve and release Al ions into the soil solution (Koenig et al. 2011; Zhou et al. 2011). The rate of dissolution of Al-bearing minerals is pH-dependent; therefore, Al ions tend to increase with decreasing soil pH (Violante et al. 2010). Aluminium can adsorb non-specifically to negatively charged sites on clay minerals and hydrous oxides of iron, aluminium and manganese via electrostatic forces (Violante et al. 2010). However, it can also adsorb specifically to hydrous oxides containing variably charged sites, as well as to the edges of clay minerals and in between layers of silicate clays.

The soluble forms of Al consist of a multitude of Al species produced from hydrolysis, and these include Al^3+^, Al(OH)^2+^, Al(OH)_2_^+^, Al(OH)_3_ and Al(OH)_4_^−^ (Nordstrom and May 1996). However, trivalent Al^3+^ tends to dominate in soils at pH < 5, while Al(OH)^2+^ and Al(OH)_2_^+^ species are formed as the soil pH increases (Violante et al. 2010). While gibbsite [Al(OH)_3_] occurs at neutral pH, aluminate [Al(OH)_4_^−^] dominates under alkaline conditions (Haynes and Mokolobate 2001; Ma et al. 2001).

Organic Al is formed when exchangeable Al binds to organic ligands in the soil to produce stable complexes (Delhaize and Ryan 1995). These include mobile and exchangeable aluminium, assimilable aluminium and Al^3+^cations in water-soluble compounds. The highest mobility of Al occurs between pH 4.0 and 4.5 (Barabasz et al. 2002). In soil, Al affects every aspect of legume N_2_ fixation, including the host plant, the rhizobia and their interaction.

## Toxicity and tolerance of aluminium in symbiotic partners

**Fig. 1:**
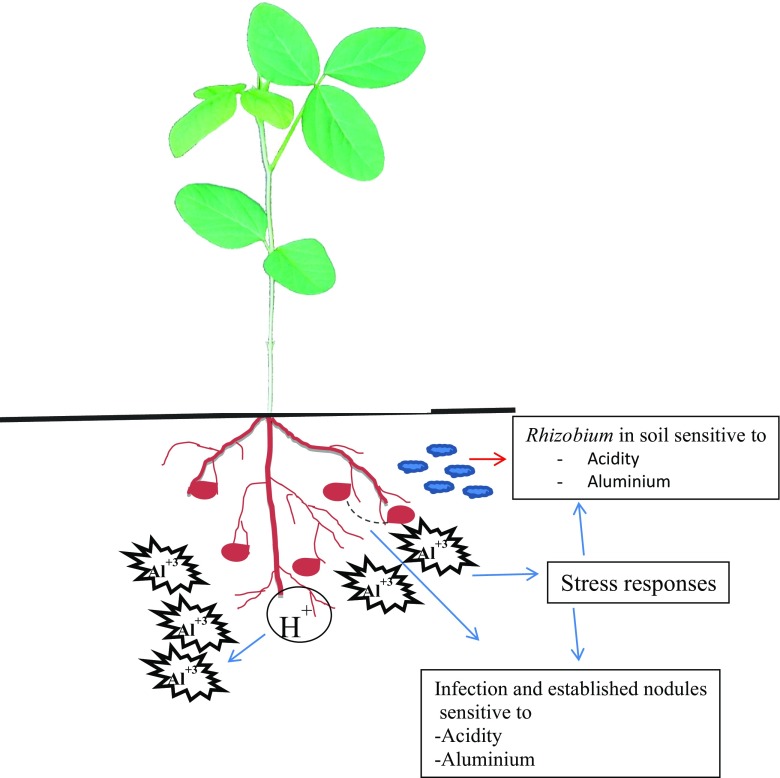
Effect of aluminium on legume nodulation under acidic conditions

Plant species differ in their response to Al. For example, Meso-American common bean genotypes have been found to be less resistant to Al than Andean common bean genotypes (Blair et al. 2009). Nodulated legumes are also reportedly more sensitive to Al toxicity than plants receiving mineral N (Hungria and Vargas 2000; see Fig. 1). Although soybean growth was decreased by 54% at 10 μM Al, rhizobial growth was inhibited at 50 μM Al (Arora et al. 2010; Kopittke et al. 2015), confirming that the microsymbiont and the infection process are less sensitive to Al toxicity than host plant growth (Table 1). Al-dependent acid pectin production can also increase cell wall thickening and rigidity of infection threads (Sujkowska-Rybkowska and Borucki 2015), leading ultimately to altered infection thread formation and nodule development. It is these subtle effects of Al that cause the commonly observed reduction in nodule number and/or complete nodulation failure in temperate and tropical legumes exposed to Al (Mendoza-Soto et al. 2015; Paudyal et al. 2007), in addition to Al suppression of *nod* gene induction in symbiotic rhizobia (Richardson et al. 1988). But the activity of the nitrogenase enzyme itself is reduced when Al accumulates in the bacteria-infected zone of root nodules (Mendoza-Soto et al. 2015). That notwithstanding, some rhizobial strains are resistant to Al (Zahran 1999), but how these resistant strains avoid suppression of *nod* gene induction by Al (Richardson et al. 1988) remains to be determined.

**Table 1:** Effect of Al concentration on rhizobia, legume and their interaction

	Nodulate	Al susceptibility (μM)	Reference
Strain			
*Bradyrhizobium* BMP1	*Mucuna pruriens*	> 100	Arora et al. (2010)
*Sinorhizobium* RMP_5_	*Mucuna pruriens*	> 50	Arora et al. (2010)
*Rhizobium* UFLA04-195, UFLA04-173, UFLA04-202	*Phaseolus vulgaris*	> 2000	Ferreira et al. (2012)
*Bradyrhizobium*	*Acacia*	> 50	Vargas et al. (2007)
Legume			
Andean *Phaseolus vulgaris*		> 25	Blair et al. (2009)
*Glycine max*		> 4.7	Silva et al. (2001)
*Pisum sativum*		> 50	Sujkowska-Rybkowska (2012)
Interaction			
Clover-*Rhizobium*		< 25,000	Jarvis and Hatch (1985)

Recently, 28 Al toxic-response miRNAs have been identified in common bean nodules (Mendoza-Soto et al. 2015). Whether this is an indication of their broader involvement in alleviating Al stress remains to be assessed. It has however been reported that miRNA target genes can code for stress-response proteins that affect plant functioning during metal toxicity (Gupta et al. 2014; Zeng et al. 2014). But again, the mechanism underlying the relief of Al stress by miRNAs is still not understood. Furthermore, we still do not know whether miRNAs also play a role in bacterial tolerance of Al toxicity.

Root secretion of Krebs cycle intermediates has been regarded as a major feature of Al tolerance in land plants. The effect of Al^3+^ on alfalfa root tips and nodules was enhanced by the synthesis of the enzymes malate dehydrogenase (MDH) and phosphoenol pyruvate carboxylase (PEPC), which catalyse the formation of carboxylic acids (Tesfaye et al. 2001). In transgenic alfalfa, Al^3+^ tolerance in root tips was greatly enhanced by the over-expression of bacterial citrate synthase in roots (Barone et al. 2008). Furthermore, the results of in vitro experiments showed that organic acids are able to reverse Al-induced conformational changes in the regulatory protein and calmodulin and restore its activity. Rhizosphere increase in pH via extrusion of hydroxyl ions by root apices is another way to precipitate Al and reduce cell damage (Delhaize and Ryan 1995). This probably explains the alkalisation in the rhizosphere of Rooibos tea legume, *Aspalathus linearis* subsp. *linearis*, when grown at pH 3 (Muofhe and Dakora 1998). Al tolerance in plants has therefore been associated with increased accumulation of Al^3+^ in the rhizosphere and roots but reduced concentration in photosynthetic shoots.

The mechanism of Al resistance in symbiotic rhizobia is much less understood relative to the host plant. Nevertheless, rhizobia can vary in their tolerance of Al (Kingsley and Bohlool 1992), and both Al-sensitive and Al-tolerant rhizobia have the potential to bind with Al^3+^ (Ferreira et al. 2012). The DNA of rhizobial strains could be a possible site of action for Al as a DNA repair mechanism appears to exist in tolerant strains of *Mesorhizobium loti* and DNA synthesis in Al-tolerant strains was not affected by Al^3+^ supply (Johnson and Wood 1990).

Richardson et al. (1988) observed a reduction in *nodA* gene expression in *Rhizobium leguminosarum* bv. *trifolii* strains at 7.5 μM Al^3+^, leading to cell death and decreased N_2_ fixation as the concentration of Al increased from 25 to 50 or 80 μM (Kingsley and Bohlool 1992). Production of exopolysaccharides (EPS) could also be a mechanism for Al tolerance in rhizobia, as tolerant strains are reported to produce more EPS than their sensitive counterparts (Ferreira et al. 2012). More studies are needed to confirm the role of EPS in rhiziobial tolerance of Al. The induction of efflux pumps is another mechanism used by bacteria to overcome heavy metal toxicity (Nies 2003). But whether these efflux pumps and protein transporters are involved in the Al tolerance of rhizobia remains to be determined. Interestingly, microsymbionts such as *Mesorhizobium metallidurans* isolated from root nodules of *Anthyllis vulneraria* can naturally tolerate high concentrations of heavy metals such as Zn (16–32 mM) and Cd (0.3–0.5 mM) (Vidal et al. 2009). But it is still unclear whether the efflux pumps and protein transporters found in heavy metal-tolerant bacteria also exist in symbiotic rhizobia for Al tolerance. Furthermore, whether the resistance of *M. metallidurans* to Zn and Cd is via efflux pumps or phytostabilisation of active ions is still unknown. However, a recent report has suggested that siderophores produced by microbes could also be involved in the protection against the toxic effect of Al by formation of siderophore-metal complex (Schalk et al. 2011). The presence of the siderophores, pyochelin and pyoverdine individually reduced the uptake of Al by 80% in Gram-negative bacteria, which include rhizobia (Braud et al. 2010). Furthermore, metal-inducible (*dme*RF) gene clusters have been discovered in *Rhizobium leguminosarum* bv. *viciae* and other members of the Rhizobiaceae that are expressed in response to heavy metal concentrations (Rubio-Sanz et al. 2013). This could suggest that the *dmeRF* gene probably plays a key role in rhizobial tolerance of metals such as Al. Additionally, studies of heavy metal resistance in rhizobia isolated from metallicolous legumes suggest that these strains have genes that encode for metal efflux systems (Teng et al. 2015).

## Effects of Al on rhizobia

Besides plants, soil microbes are also adversely affected by moderate to high levels of exchangeable Al present in acidic soils (Ferreira et al. 2012; Paudyal et al. 2007). High Al^3+^ concentration can be detrimental to N_2_-fixing bacteria whether in soil or culture medium (Arora et al. 2010; Ferreira et al. 2012; Kinraide and Sweeney 2003; Rohyadi 2006) through changes in cellular metabolism that affect bacterial growth and survival. Acid tolerant (pH < 5.0) rhizobia (CIAT899, UFLA04-195, UFLA04-122, UFLA04-202, UFLA04-173, UFLA04-155, UFLA04-226, UFLA04-228, UFLA04-229, UFLA04-231, UFLA04-233, UFLA04-232 and UFLA04-21) grew at 500 μM of Al^3+^ (Ferreira et al. 2012; Graham et al. 1994). According to Roy and Chakrabartty (2000), about 35% reduction in rhizobial cell mass occurred in media with 1 Mm (1000 μM) Al relative to control. In one study, *Sinorhizobium meliloti* strain RMP_5_ was more tolerant of Al than *Bradyrhizobium* BMP_1_; the former could therefore grow at more than 100 μM Al concentration (Arora et al. 2010). Whatever the case, it appears that where there was sensitivity to added Al, enzymatic function of nitrate reductase, nitrite reductase, bacterial nitrogenase and uptake hydrogenase was impaired by Al in both slow- and fast-growing rhizobia. However, in another study, the growth of all test rhizobia was impaired by 25 to 100 μM Al concentration (Paudyal et al. 2007). Common bean-nodulating rhizobia isolated from an Amazon soil containing > 2 mM (> 2000 μM) Al showed retarded cell multiplication (Ferreira et al. 2012). In contrast, Vargas et al. (2007) found no effect of 50 μl Al^3+^ L^−1^ on the growth of ten *Acacia*-nodulating isolates from south Brazil. There is no well-defined mechanism reported for acid-tolerant in bacteria yet. However, several reports have suggested that this tolerance is due to their maintaining of a consistent cytoplasm pH, differences in lipopolysaccharide membrane composition and proton’s exclusion, polyamine accumulation and modification in membrane lipids (Chen et al. 1993; Ferreira et al. 2012).

## Effect of Al on the legume/rhizobia symbiosis

The outcome of interaction between rhizobia and legumes depends not only on the bacterium and the plant species, but also on the soil supporting the growth of the symbiotic partners (Ferreira et al. 2012). The early stages of the legume/rhizobia symbioses are very sensitive to low pH and high Al concentration, as they can both affect *nod* gene expression, Nod factor production and hence nodule formation (Abd-Alla et al. 2014). Inhibition of nodulation due to high Al concentration has been reported for several legumes, including *Phaseolus vulgaris, Trifolium repens, Stylosanthes* species and other tropical species (Mendoza-Soto et al. 2015; Paudyal et al. 2007). As a result, acid tolerance in a legume may not necessarily guarantee greater yield in acidic soils because bacterial multiplication and survival in soils are highly affected by the combined effect of acidity and Al. Both the interaction and host plant growth per se are reduced by Al concentrations as low as < 25 mM m^−3^ (< 25,000 μM m^−3^) (Jarvis and Hatch 1985; Wood et al. 1984). Both rhizobial growth and legume root infection are restricted by low pH as well as Al toxicity associated with acidic soils (Ferreira et al. 2012; Paudyal et al. 2007). In fact, Al inhibition of rhizobial infection, root hair curling and nitrogenase activity have been known for a long time (Ayanaba et al. 1983; De Manzi and Cartwright 1984; Munns 1978; Munns et al. 1979; Wood et al. 1984). High levels of Al can therefore reduce rhizobial populations in soil, thus impairing the BNF process (Barabasz et al. 2002). Nitrogen deficiency can easily develop in legumes as a result of Al inhibition of nodule formation. The presence of Al^+3^ reduces Ca uptake during symbiotic process of nitrogen fixation (Andrew 1976; Munns 1970). As a result, delayed nodulation has been linked to Al toxicity in acid soils with low Ca concentrations (Schubert et al. 1990). Therefore, rhizobial inoculants are likely to have a lower chance of success in acidic soils with high Al concentration (Roy and Chakrabartty 2000). In another report, Goedert (1983) and Sprent et al. (1996) have found that certain legumes in Brazil savanna are capable of nodulating and fixing N_2_ in soils with high Al. Many *Lupinus* species and native soil rhizobia in the Mediterranean regions are naturally resistant to low pH and high Al concentration (Sprent 2009); such symbioses can therefore be selected for use in the world’s acidic soils.

## The *Aspalathus linearis* symbiosis: a natural system for understanding Al tolerance in perennial legumes and their microsymbionts

**Fig. 2:**
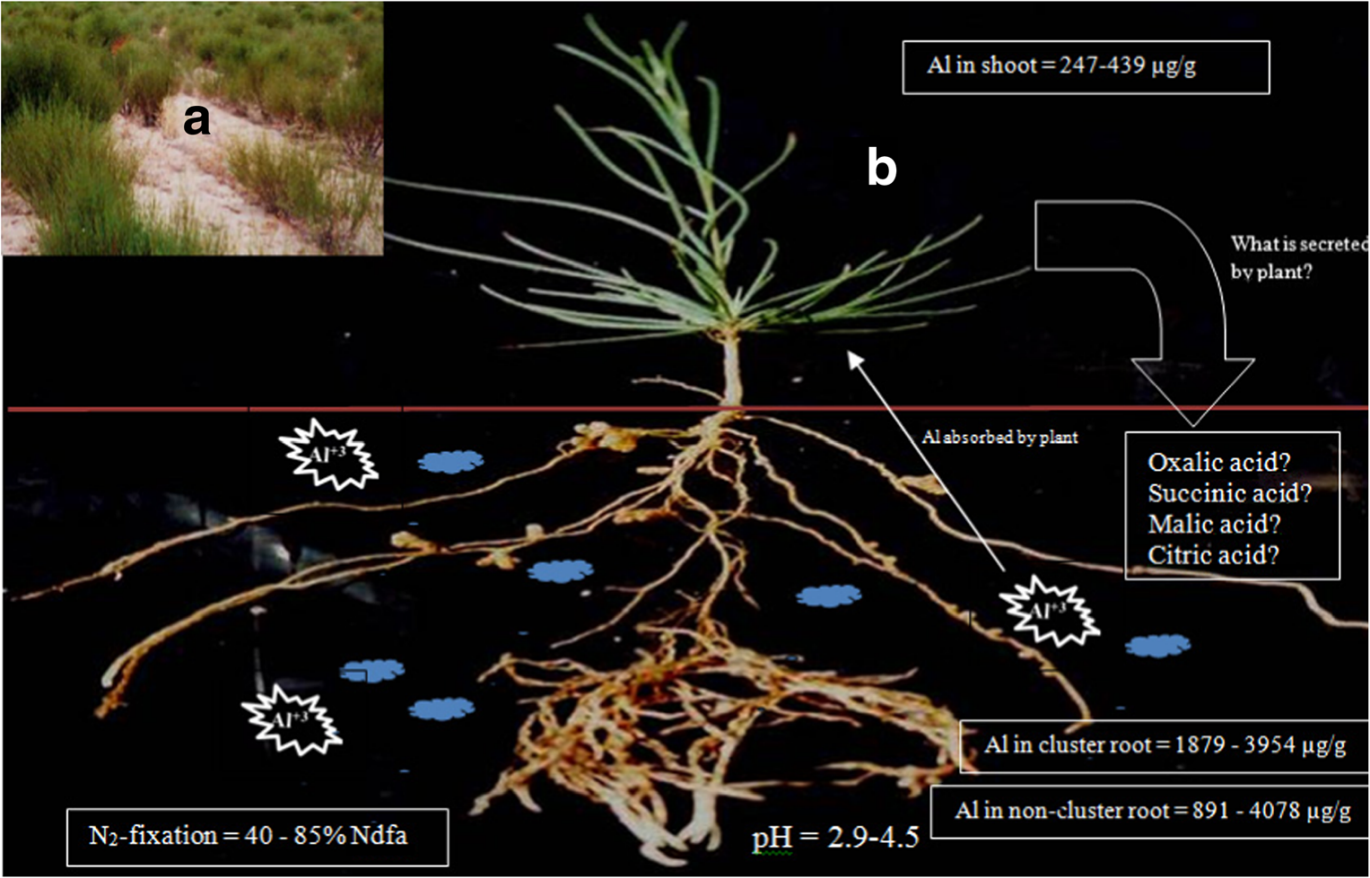
**a** *A. linearis* plants growing in the field in a sandy acidic nutrient-poor soil. **b** Nitrogen fixation and concentration of Al in clustered root, non-clustered root and shoot of *A. linearis*

*Aspalathus linearis* subsp. *linearis* grows naturally in the ecosystem, as well as a cultivated plant in farmers’ fields in the sandy, highly acidic, Al rich soils of the Cape Fynbos in South Africa. This legume is the source of ‘Rooibos tea’, a health tonic that contributes substantially to the agric GDP of South Africa. *Aspalathus linearis* is nodulated by *Bradyrhizobium*, *Mesorhizobium* and *Burkholderia* species (Hassen et al. 2012). As shown in Fig. 2, this legume and its rhizobia are capable of growing in acidic, Al-rich soils with pH 2.9 to 4.5 (Muofhe and Dakora 1998). Surprisingly, they can meet as much as 40 to 85% of their N requirements from symbiotic fixation under those stressful abiotic conditions (Muofhe and Dakora 1999; Fig. 2). Here, we propose mechanisms for the ability of *A. linearis* and its microsymbionts to survive and fix abundant N_2_ under those harsh environmental conditions. Firstly, this legume is reported to secrete hydroxyl ions which increase rhizosphere pH from pH 2.9 to pH 6.6 (Muofhe and Dakora 2000). In doing so, rhizobial infection and root nodulation can occur under less harsh optimal pH conditions. Secondly, we have found that although the levels of endogenous Al can be quite high in soils supporting the growth of *A. linearis*, the Al concentration in shoots is very low relative to those in below-ground organs such as cluster roots and non-cluster roots (Dakora et al. unpublished data). We postulate that organic acids (OAs) secreted by roots and cluster roots chelate with active Al to form inactive complexes in the rhizosphere. We also suggest that these OAs inside roots and cluster roots form complexes with incoming active Al ions to form inactive Al-OA complexes that are stored in non-toxic forms in roots and cluster roots. This model could explain why the Al concentrations in below-ground organs such as roots and cluster roots are many folds greater than Al levels in above-ground shoots. In our view, this constitutes the mechanism by which *A. linearis* can thrive in Al-rich, highly acidic soils in the Cape Fynbos of South Africa. Taken together, these biochemical subtleties in Al tolerance support *A. linearis* as a natural system for studying metal tolerance in nodulated perennial legumes (Table 2).

**Table 2:** Effect of soil aluminium on legumes, their microsymbionts, nodule formation and nitrogen fixation

Effect of Al^+3^ toxicity on plants	Reference
Prevent toxic effect of Cu and Mn	Barabasz et al. (2002)
Protect plant from fungi, extreme temperature and soil salinity	
Suppress nodulation	Rohyadi (2009); Zhou et al. (2011)
Reduced elongation in root hairs	
Failure of root hair formation	
Reduced nutrient and water uptake	Haynes and Mokolobate (2001); Zhou et al. (2011)
Reduced nitrogen fixation	Jarvis and Hatch (1985); Silva and Sodek (1997)
Reduced rhizobial cell mass	Wood et al. (1984); Whelan and Alexander (1986) Barabasz et al. (2002); Arora et al. (2010)
Reduced symbiotic relationship between legume and rhizobia	Blamey et al. (1983); Jarvis and Hatch (1985); Lesueur et al. (1993)
Inhibit curling of root hair	Ayanaba et al. (1983)
Inhibit nitrogenase activity	De Manzi and Cartwright (1984); Mendoza-Soto et al. (2015)
Inhibit cell division	Wood (1995); Frantzios et al. (2005)
Inhibit hexokinase, acid and alkaline phosphatase, phosphodiesterase and phosphooxidase	Bennet and Breen (1991); Barabasz et al. (2002)
Reduced root growth	Rengel and Robinson (1989); Kopittke et al. (2015); Mendoza-Soto et al. (2015)

Furthermore, the ability of legumes such as *Aspalathus linearis* to accumulate Al in mainly roots with very little translocated to shoots has great potential for phytoremediation which can be exploited for the ecological economy of degraded ecosystems. Some of the environmentally safe and microbially based bioremediation approaches that can be tapped for ecosystem mangement include (i) the selection and use of legume/rhizobia symbioses resistant to metals, (ii) the use of mixed inoculants containing metal-resistant rhizobia and plant growth-promoting rhizobacteria and (iii) plant inoculation with a mixture of rhizobia and mycorrhizae (Pajuelo et al. 2011). For example, the combined use of Cd-tolerant rhizobacteria (Siripornadulsil and Siripornadulsil 2013) and Cr-resistant plant growth-promoting bacteria isolated from contaminated soils (Rajkumar et al. 2006) has great potential for land reclamation and phytoremediation of degraded natural ecosystems.

Interestingly, while there is evidence of acid-tolerant genes in symbiotic rhizobia (Dilworth et al. 2001; Glenn et al. 1999; Laranjo et al. 2014) that permit bacterial survival in Al-rich and low-pH soils supporting growth and N_2_ fixation of *A. linearis* (Muofhe and Dakora 1999), little is known about Al-tolerant genes in legumes and their microsymbionts. This is perhaps not unexpected as no crop species are yet known that tolerate high concentrations of Al in soils. Given the many acidic soils in the world that are already heavily loaded with high level of Al, future studies must identify genes in both legumes and rhizobia that control Al toxicity in the two symbiotic partners. That way, food/nutritional security and environmental health would be assuredly enhanced.

## Amelioration of Al toxicity

Al phytotoxicity can be amended through liming with calcium carbonate, addition of organic matter and/or by use of Al-tolerant species (Mokolobate and Haynes 2002). Liming stimulates soil organic carbon mineralisation by increasing soil pH and detoxification of Al and increases microbial survivability by C use efficiency (Grover et al. 2017; Wang et al. 2016). Liming with Ca can alleviate Al toxicity through enhancing the ionic strength of the soil solution and thus increasing competition between Al and Ca for binding sites of cell membranes (Kinraide and Parker 1987). Addition of Ca to an acidic sub-surface solution in a vertically split root system for different soybean genotypes resulted in an improved rooting system (Ferrufino et al. 2000). The Ca/Al activity ratio of 891 genotypes caused a 50% reduction in tap root length. However, lateral roots required a greater concentration of Ca^2+^ to overcome inhibition of root elongation by Al. Thus, even though tap roots might extend into acidic soil zones, development of lateral roots for nutrient and water capture could still be limited (Ferrufino et al. 2000). More Ca was needed in Al-sensitive genotypes to offset the toxic effects of Al on root elongation (Silva et al. 2001).

Liming has also been found to increase Ca availability to rhizobia and the symbiosis (Hungria and Vargas 2000). However, this practice is not economically feasible (Foy 1988), especially for small-scale subsistence farmers and may also not be cost-effective in sub-soils due to poor Ca distribution during tillage (Gourley 1987). Rhizobial and legume response to Ca supply can also be limited by high H^+^ and Al^+3^ activities (Sanzonowicz et al. 1998). Furthermore, Al effect on soybean root elongation was countered by 10–50 μM Mg in culture solution where Al had inhibited root extension (Silva et al. 2001). Here, the Mg probably detoxified Al by reduction of Al ^+3^ activity at root cell plasma membrane, thus preventing the disruption of cell expansion and cell division commonly induced by Al toxicity (Kochian 1995). Similarly, the beneficial effect of Si on Al toxicity has been reported for soybean (Baylis et al. 1994). Applied Si can form hydroxyaluminosilicate complexes with Al in the external soil solution and thus render the Al ions inactive and non-toxic to both plants and rhizobia (Pontigo et al. 2015).

## Organic matter amendment

Organic matter can also be used to overcome Al toxicity in plants and microbes (Foy 1984, 1988; Rohyadi 2006). During decomposition of animal and plant debris, a whole range of organic compounds released by soil microbes combine with active Al ions to form complexes that are non-toxic to both plants and rhizobia (Haynes and Mokolobate 2001; Suthipradit et al. 1990). Furthermore, adding organic residues to soils often results in an initial increase in soil pH, which can potentially decrease exchangeable Al in the soil and thus reduce its phytotoxicity (Haynes and Mokolobate 2001).

## Conclusion

Taken together, Al stress is a major abiotic factor affecting plant growth and productivity. With 40% of the world’s arable land consisting of acid soils and Al toxicity being associated with low pH, global legume production is likely to be hugely constrained. This is because Al toxicity in soils can inhibit root elongation, lateral root development, root hair growth, rhizobial infection of the roots, Nod factor production and nodule development, resulting in low N_2_ fixation and decreased crop yield. Therefore, selecting legume/rhizobia symbioses that are tolerant of Al toxicity is the easiest way to increase crop yields in Al-rich acidic soils. A better understanding of legume exudation in response to Al toxicity and the mechanisms underlying rhizobial tolerance of Al stress is crucial for increasing yield of grain and pasture legumes. Furthermore, understanding gene expression in the presence of added Al may be a strategy for identifying rhizobial genes and legume traits that permit high N_2_ fixation in the presence of Al stress.
